# Graphical Visualization Approach for Two-Dimensional Liquid Chromatography with Parallel Column Arrays

**DOI:** 10.1021/acsmeasuresciau.6c00035

**Published:** 2026-04-06

**Authors:** Deklin Parker, Samuel W. Foster, Tina Dahlseid, Dwight R. Stoll, James P. Grinias

**Affiliations:** † Department of Chemistry & Biochemistry, 3536 Rowan University, Glassboro, New Jersey 08028, United States; ‡ Department of Chemistry, 7573 Gustavus Adolphus College, Saint Peter, Minnesota 56082, United States

**Keywords:** 2D-LC, Parallel Column Arrays, Data Visualization, Python, Multidimensional Chromatography

## Abstract

Two-dimensional liquid chromatography (2D-LC) is one of the highest-resolution techniques available to chromatographers for the separation of analytes in complex mixtures. However, data analysis and data visualization remain challenging, especially for home-built systems that do not utilize commercial software packages. Even these packages are not capable of effectively displaying data generated from systems where multiple columns are used for the second-dimension separation. Here, an open-source software package with a graphical user interface used to plot and characterize 2D-LC data is described. In addition to plotting imported data, a 2D-LC simulator is included to generate both 1D and 2D chromatograms. The HSV (hue, saturation, value) color wheel is used to combine multiple 2D-LC contour plots into a single graphic, creating a new visualization strategy for 2D-LC systems in which multiple columns are used sequentially or in parallel column arrays in the second dimension. Use of the package for both experimental and random simulated data is presented as a demonstration of its capabilities.

## Graphical abstract

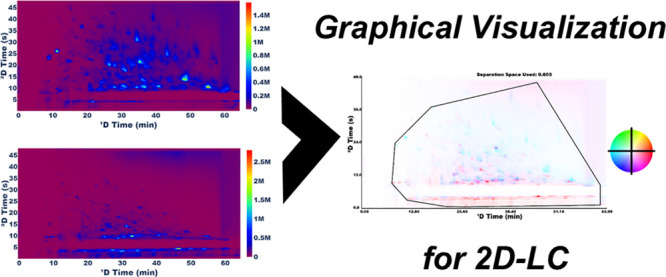

## 1 Introduction

Two-dimensional liquid chromatography (2D-LC) is continuing to grow as an important analytical methodology for the separation of complex mixtures.^1,2^ It has found utility in the analysis of pharmaceutical compounds,^3^ polymers,^4^ proteins,^5^ and a wide variety of other applications.^6^ By utilizing two complementary separation modes in a single method, the overall peak capacity can be increased, improving the quality of the separation and the capability to determine more analytes in the mixture.^7^ Multiple columns operated in parallel arrays can be used to increase the selectivity of the 2D-LC method by expanding the number of stationary phases that are used in the second-dimension separation either simultaneously (with split injections) or in sequential, consecutive operation with multiple runs.^8^ Parallel column arrays in the second dimension can also be used to decrease the cycle time of the second-dimension separation when operated in a sequential injection approach.^8^ Typically, 2D-LC chromatograms are depicted as two-dimensional contour plots with each axis representing the retention time in one column dimension and color variation used to indicate signal intensity. This approach is limiting when using parallel column arrays (sequentially or simultaneously) in the second dimension, as a separate plot is needed to represent the separation on each individual column.^9^ To better visualize the data produced in these experiments, a custom, open-source graphical visualization package is described here that enables the combination of up to four 2D-LC contour plots (equivalent to a second-dimension array of up to four columns) in a single graph using vector addition and a HSV color wheel (hue, saturation, value).^10^ This builds upon existing chemometrics approaches using the RGB color model to simplify spectroscopic characterization in image analysis.^11^ A 2D chromatogram simulator using random peak generation is included, providing the opportunity to test the software package with both simulated data and imported raw experimental data files. By utilizing open-source software design principles,^12^ this application provides a useful tool for characterizing and visualizing 2D-LC data obtained with both home-built and commercial instrumentation that allows implementation with more than one column in the second dimension.

## 2 Software Design and Approach

The 2D-LC simulator and graphical visualization software package (available in Supporting Information (SI) section SI2) was constructed in Python, based upon open-source libraries described in Table 1. A number of LC simulation tools are available online,^13,14^ but most focus on unidimensional separations and fewer are available for multidimensional separations.^15,16^ Here, a 2D chromatogram simulator with randomized peak placement was developed as a tool for users and to provide a simple way of generating *in silico* data that could be used to test the graphical visualization tool. A general flowchart of the program operation is shown in Figure 1 and a full description of its use can be found in the SI. For isocratic separations in the first dimension, a random retention time (*t*_r_) for each peak is generated between the void time and the maximum analysis time (in this case 1000 s). This method of random generation follows the Poisson distribution.^17^ From these retention times, a peak width (*w*_B_) is calculated based on the user defined column efficiency (in plate count, *N*) as follows:^18^

1 $$w_{B}=4\sigma_{t}=\frac{4t_{r}}{\sqrt{N}}$$

A peak is then generated as a normal Gaussian distribution based on the following equation:^19^

2 $$f\left(t\right)=\frac{1}{\sqrt{2\pi {\sigma_{t}}^{2}}}e^{-{\left(t-t_{r}\right)}^{2}/2{\sigma_{t}}^{2}}$$

The distribution is scaled along the *y* axis to a randomly generated peak and then added to the baseline. This process is repeated until the user-selected total number of peaks has been generated.

**Figure 1:**
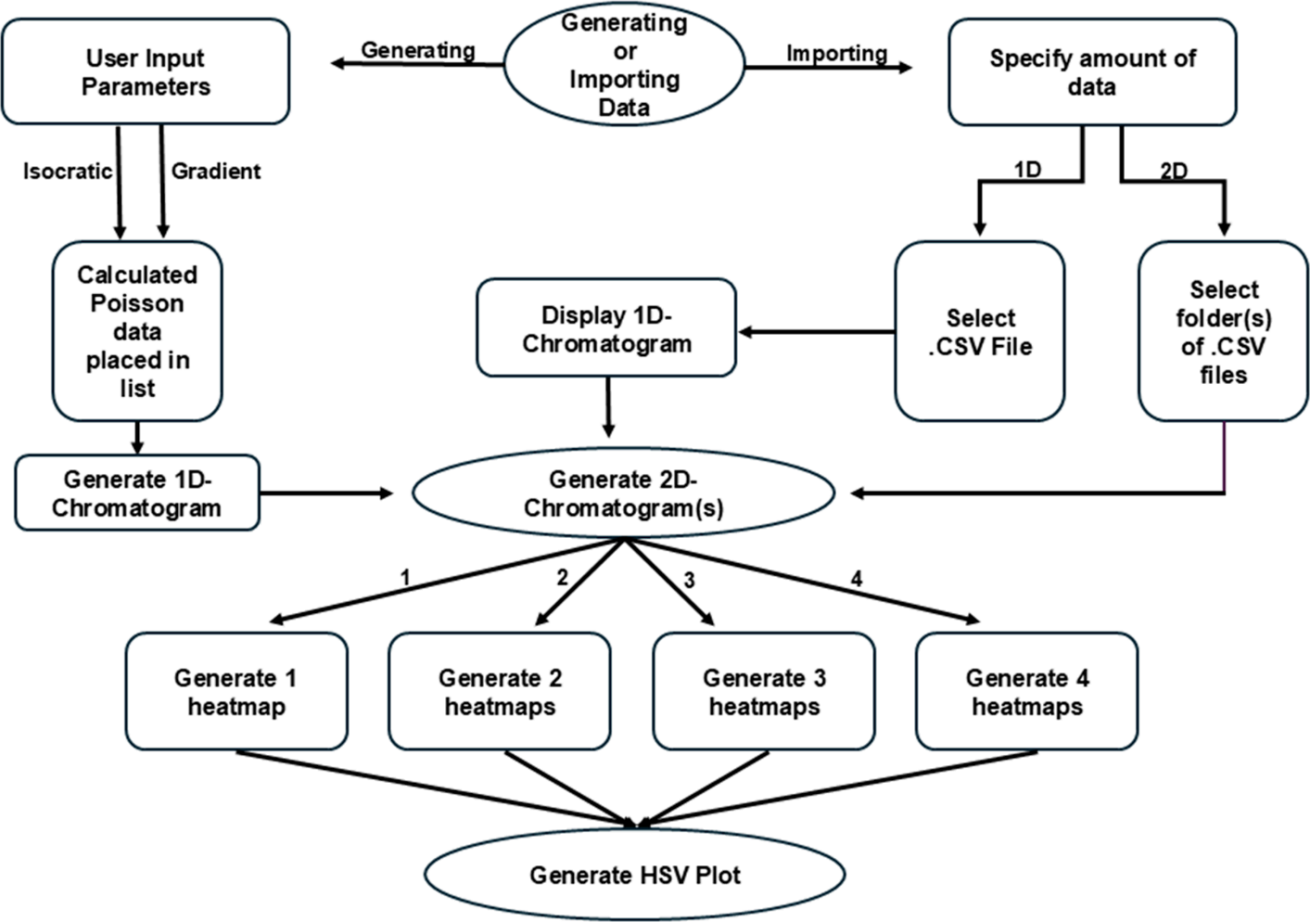
Logic flowchart for operation of the open-source simulation and plotting software.

**Table 1:** List of Python Libraries Used in Development of the Graphical Visualization Program

library	purpose	online location	ref
tkinter	value input and file select	https://docs.python.org/3/library/tkinter.html	^29^
matplotlib	first-dimension plot	https://matplotlib.org/	^30^
plotly	single-column second-dimension plots	https://plotly.com/python/	^31^
kivy	drawing and display of parallel dimension plot	https://kivy.org/	^32^
scipy	the convex hull method within the library takes in parallel dimension data and creates vertex points for calculation of complementarity	https://scipy.org/	^33^

For isocratic separations in the second dimension, the peak area (*A*) for each first-dimension fraction is calculated. Peak areas for each second-dimension peak are then randomly generated such that the total summed area of all peaks (a randomly generated number, with shape also calculated using Equations 1 and 2) in a given second-dimension separation is equal to the first-dimension area for that fraction. For these second-dimension peaks, instead of randomly generating peak height (*h*), *h* is calculated to produce peaks of their generated area using an adapted triangular estimation of peak area^20^ to simplify calculations in the program:

3 $$h=\frac{2A}{w_{B}}$$

This process is repeated for each fraction of the first-dimension effluent, generating the multidimensional dataset. Given that second-dimension peak locations are randomly generated, and that their peaks represent a random distribution of a fixed area amount, multiple second-dimension chromatograms can be generated and easily compared to one another.

To generate a simulated gradient separation in the first dimension, it is assumed that peak widths across the gradient are uniform based on linear solvent strength theory.^21^ Similar to the isocratic separation generation, the retention times are determined via Poisson distribution and the widths are determined by the calculated width of the first peak in the separation. Using Equation 2, peaks of identical width are generated around each calculated retention time before being scaled to a random height. For gradient separations in the second dimension, a similar method utilizing a Poisson distribution of retention times and eq 2 is used to generate the second-dimension separations. The peak area from the first-dimension fraction is calculated and randomized peak areas are generated. The peaks are then spread equally over the separation space such that all have the same width. Equation 3 is then used to calculate the height of each peak. This process is repeated for each second-dimension separation, generating a full second-dimension plot.

**Figure 2:**
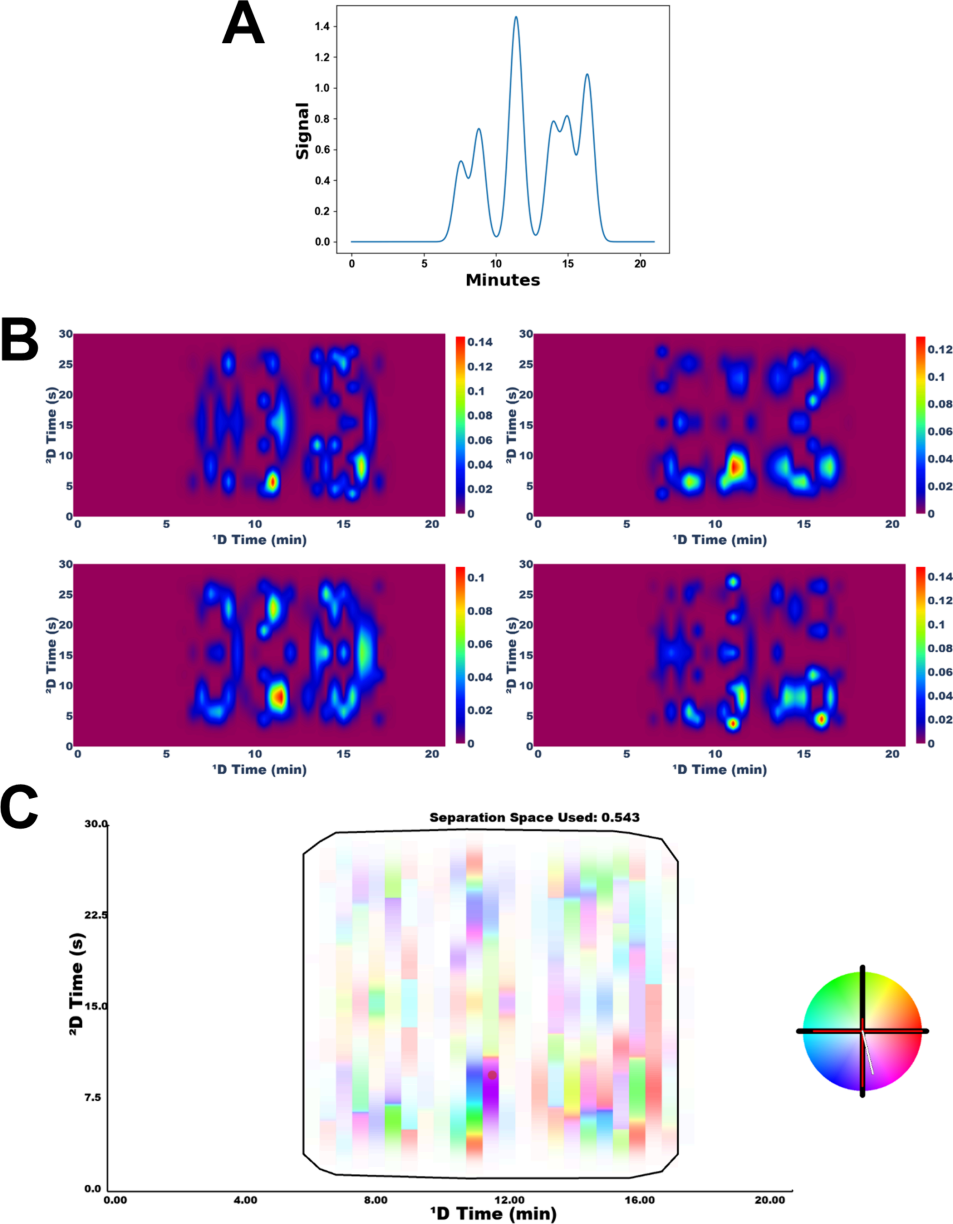
Generation of a simulated unidimensional chromatogram (A) and four separate 2D chromatograms simulated based on the first dimension assuming a parallel array of four columns (B). A combined contour plot with combinations based on HSV color integration is shown in (C), with the dot corresponding a selected point that is then shown as a white vector on the color wheel to the right of the contour plot.

**Table 2:** Comparison of Average Complementarity for 2D-LC Separations Using One to Four Columns in the Second Dimension^a^

	complementarity			
number of simulations	*N*_c_ = 1	*N*_c_ = 2	*N*_c_ = 3	*N*_c_ = 4
50	0.60 ± 0.14	0.66 ± 0.16	0.67 ± 0.16	0.68 ± 0.17
100	0.58 ± 0.15	0.63 ± 0.16	0.64 ± 0.16	0.66 ± 0.17
1000	0.59 ± 0.15	0.64 ± 0.15	0.66 ± 0.15	0.67 ± 0.16

a Complementarity values are presented as average ± 1 standard deviation. *N*_c_ is the number of columns in the second dimension.

**Figure 3:**
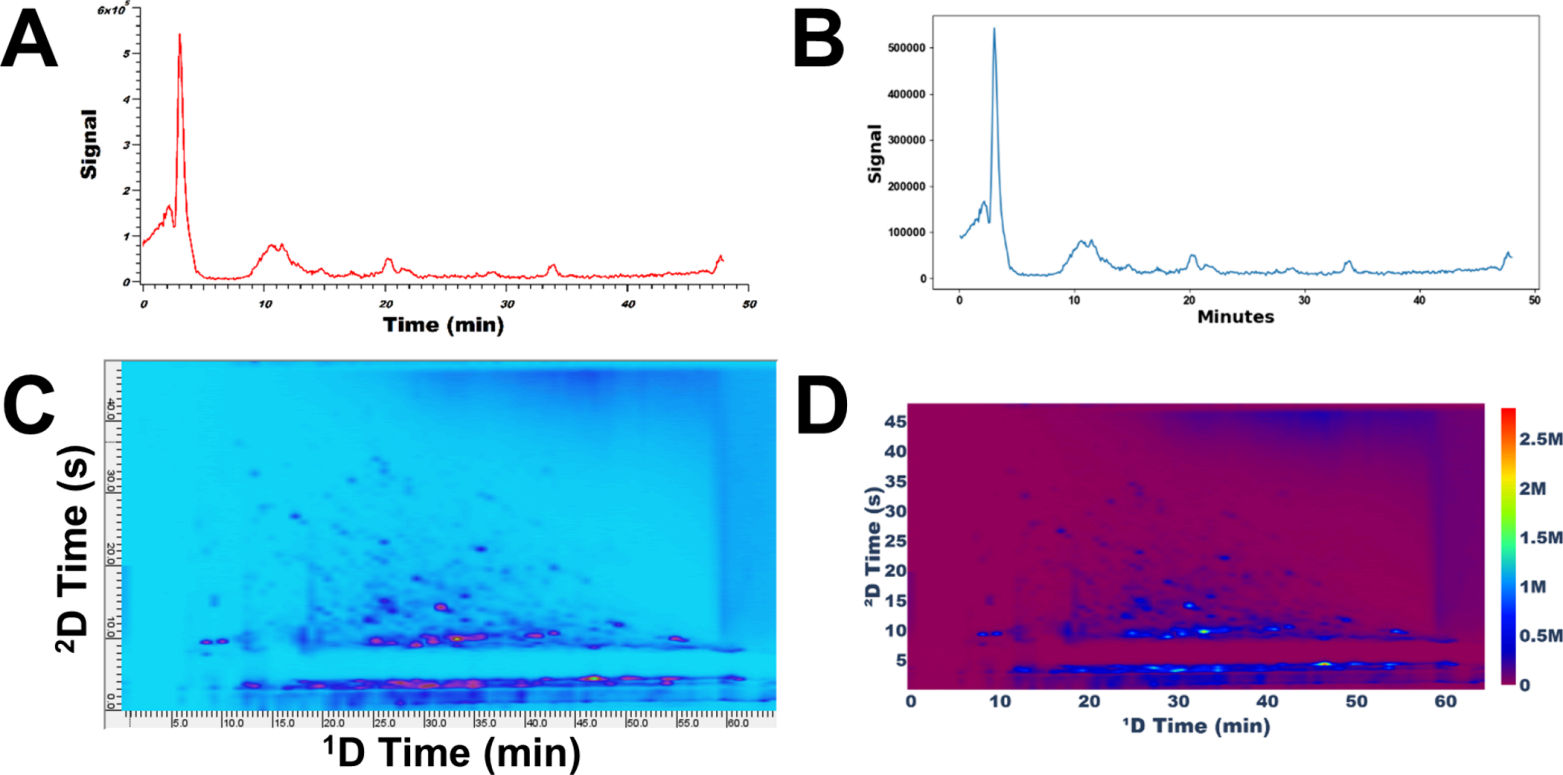
Comparison of commercial CDS (A and C) and open-source plotting software described here (B and D) for unidimensional chromatograms (A and B) and 2D contour plots (C and D). All data from the separation of *Escherichia coli* digest were collected using a HPH-C18 column for the first dimension and an Agilent Zorbax Bouns-RP column in the second dimension. The extracted ion chromatogram was selected for peaks detected in the *m*/*z* 600–650 range, and total ion counts for this range are reported as the signal. Further information on experimental details can be found in SI section SI1.

**Figure 4:**
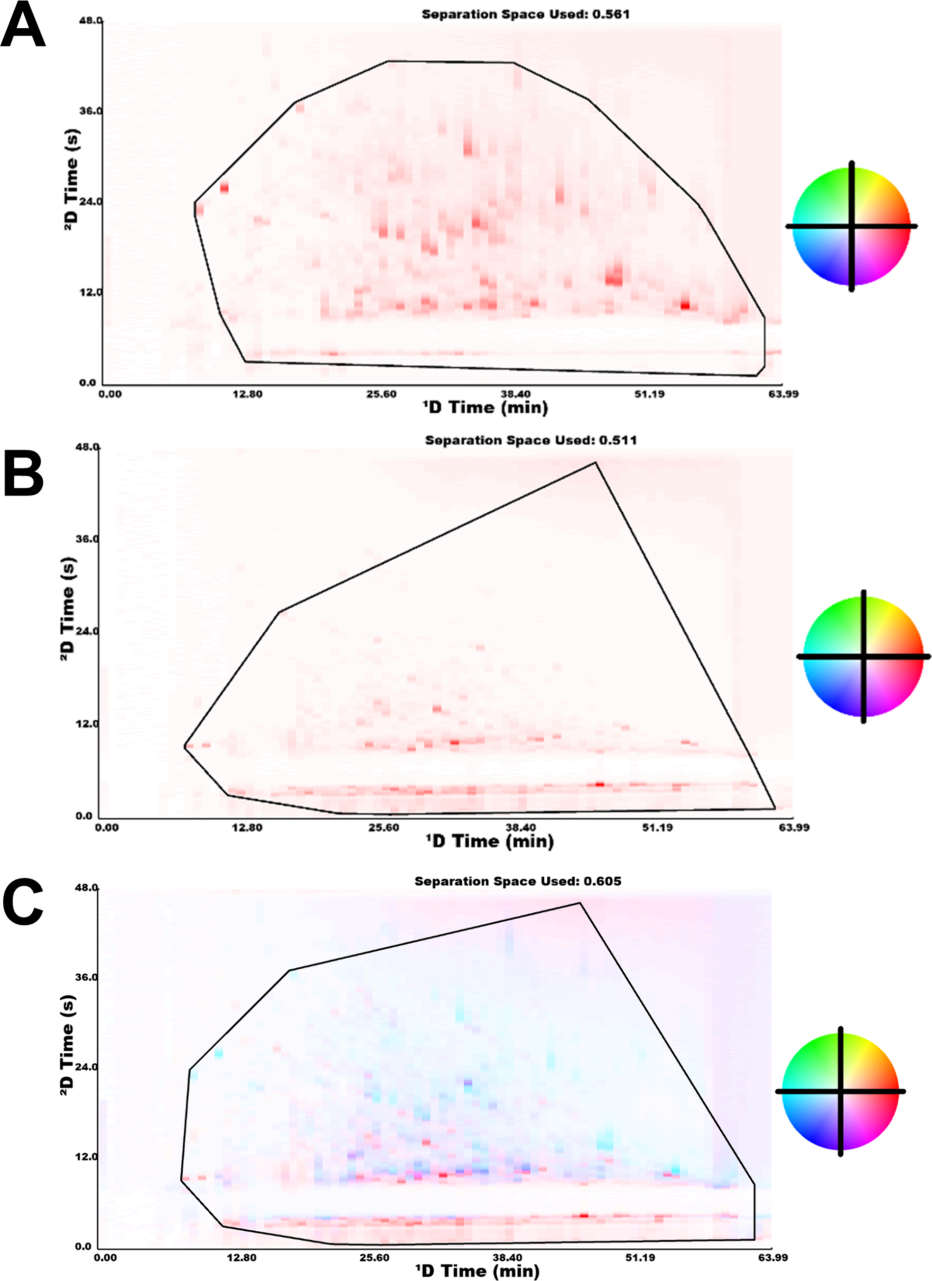
2D-LC contour plots from the separation of an *Escherichia coli* digest using a HPH-C18 column in the first dimension and either a C18 (A) and Bonus RP (B) column in the second dimension. The combined overlay plot is shown in (C). Full experimental details are provided in SI section SI1.

User-input variables include the number of peaks to include in the separation window, the chromatographic efficiency of the separation (plate count, *N*), the duration of the second-dimension separation for a comprehensive 2D-LC analysis, and the void time of the first-dimension separation. For 2D-LC simulations, the number of columns used in the second dimension can be set to one for standard operation, or increased up to four if simulating a separation with a column array in the second dimension.^8^ There is also the ability to import raw experimental data (as comma-delimited *.CSV files) to generate the plots. Unidimensional data are readily plotted as a standard signal versus time chromatogram. For 2D-LC contour plots, the peaks are smoothed by bilinear interpolation using the “plotly” library (Table 1).

If multiple columns are selected in the second dimension, a combined 2D contour plot is created in addition to the individual 2D contour plots. The combined plot of multiple second-dimension separations uses a polar coordinate system in which each dimension is set on an axis. The first dimension starts at 90°, with each subsequent dimension adding another 90°. For both experimental and simulated data, the time of the second-dimension separation must be the same for each second-dimension column. An HSV color wheel is mapped to these coordinates, allowing for vector addition to calculate the resulting color. The height value at any given point across the dimensions is added together for the saturation value. The hue is computed using the arctangent calculation from the *y*-axis values and *x*-axis values. This creates a helpful visualization of multiple second-dimension separations by demonstrating the combined contributions of each stationary phase. HSV is preferred to the RGB color system because it is easier to depict signal intensity contributions from individual columns when combining into a single point on the combined 2D contour plot. Individual data breakdowns are shown on the color wheel by clicking on a desired peak of interest; the part of the contour plot that is clicked is designated with a dot that appears on the color wheel. An overview of the process is shown in Figure 2, and a full operation guide is provided in SI section SI2.

## 3 Software Use for Visualization of Experimental and Simulated Chromatograms

The workflow in Figure 2 depicts how both simulated and experimental chromatographic data are plotted, converted into 2D-LC contour plots, and overlaid with HSV color combination for cases where multiple columns are used in the second dimension. An important aspect of determining the complementarity (often referred to as orthogonality, although complementarity is preferred to avoid problems with the specific mathematical definition of orthogonality^7^) of two techniques is characterizing the usage of the two-dimensional separation space.^22^ Here, the convex hull approach^23,24^ is used to calculate the fractional use of the two-dimensional separation space as it does not rely upon a user-defined bin size like other common methods.^7^ The convex hull for a chromatogram is established by drawing a polygon around the peak pattern where each vertex of the polygon is colocated with a chromatographic peak on the periphery of the distribution. This approach does not involve discrete bins, and thus is not sensitive to bin size, but can be sensitive to the location of outlying peaks.^23^ To determine a noise threshold, the average and standard deviation of the baseline signal of the chromatograms plotted in the contour plot is calculated. The sum of these two values determines the cutoff for noise in the data. Data less than this sum are treated as a 0, and the modified dataset is sent to the library where a polygon is drawn from the matrix along the periphery of the distribution.

The simulation of 2D-LC chromatograms using this program can be used to determine the potential increase in complementarity as the number of columns in the second dimension is increased. Here, the convex hull as a measure of the usage of the two-dimensional separation space. Using a modified version of the simulator program that enables a single randomly generated first-dimension separation to be then further fractionated into second-dimension chromatograms for one to four second-dimension columns, the complementarity for different sized parallel column arrays in the second dimension can be assessed. As shown in Table 2 for simulations of 50, 100, and 1000 random separations, there is a slight increase in complementarity as the number of columns in the array increases. The biggest increase is observed when moving from one second-dimension column to two, with diminishing gains predicted for three and four columns in the array. One limitation of this simulation involving random peak distributions is that in a standard experimental design, columns would intentionally be selected to try and maximize complementarity rather than have random retention patterns, and thus it is likely that the benefit may be slightly greater than that predicted here. Future work to expand the simulation and include this consideration could involve the application of predicted retention utilizing the hydrophobic subtraction model,^25,26^ the HPLC Column Selectivity Database,^27^ and various other retention modeling approaches.^28^

For simple plotting of experimental data, both traditional unidimensional and two-dimensional chromatograms can be generated. Figure 3 shows a comparison between the plots generated using the software package described here and a commercial chromatographic data system (CDS). The graphics obtained using both approaches are nearly identical, with only minor cosmetic differences existing due to color options between the commercial and open-source packages. This demonstrates the use of the package as a simple-to-implement open-source software tool for plotting 2D-LC data generated on either home-built or commercial systems with direct calculation of the usage of the separation space by convex hull. Direct calculation of this coverage is especially helpful when trying to explore the expansion of columns used in the second dimension to increase overall separation orthogonality. Here, this approach was examined using two consecutive 2D-LC separations of the same sample (experimental details provided in SI section SI1) in which the first column was the same in both analyses, but the second column was changed (Poroshell C18 and Bonus RP, respectively). Figure 4 shows the visualization of a 2D-LC separation with two columns in a second-dimension parallel array operated in sequential order. For visualization purposes, red is selected to represent the first column (Poroshell C18) and blue is selected to represent the second column (Bonus RP). At points of overlap in Figure 4C, the combined intensity of eluted peaks from both columns is represented with a combined purple hue. The intensity in which these colors are seen (i.e., “redder” or “bluer”) is determined by the sum of the normalized heights of the two datasets. When scaling this process up to four second-dimension phases, seen in Figure 2, the color overlap requires use of the full color wheel. The color chosen is based on the contributions of each column and the intensity of the individual contributions (found using vector addition) along the axes of the color wheel seen within the figure. As shown in Figure 4, the overlay of the two individual 2D chromatograms in a combined plot demonstrates an increased calculated area coverage relative to each individual column (0.605 vs 0.561 or 0.511, respectively), demonstrating an increase in complementarity when using multiple columns compared to a single column. Although in this experiment, the two second-dimension columns had similar retention patterns and there was not a drastic increase in complementarity, the capability to assess the difference that can be achieved with multiple columns is achieved with this open-source program.

## 4 Conclusions

An open-source data plotting software package for characterizing 1D- and 2D-LC chromatograms has been presented here, providing a tool for easily visualizing acquired data without the need for a commercial CDS. Both simulated (randomly generated within the program) and experimental data can be plotted. The calculation of fractional use of the separation space calculated by the convex hull method provides an estimate of the complementarity of the two separation dimensions, and this is expanded here to accommodate experiments that utilize parallel column arrays (simultaneous or sequential injections) in the second dimension.

## Supplementary Material





## Notes

Instructions for utilizing the graphical visualization described in the article (PDF)
Code needed to generate the Python file that can operate the program (TXT)
