# Supplementary material for: Graphical Visualization Approach for Two-Dimensional Liquid Chromatography with Parallel Column Arrays
Source: ACS Meas Sci Au. 2026 Apr 6;6(3):615–22. doi: 10.1021/acsmeasuresciau.6c00035 (PMC13281194; doi:10.1021/acsmeasuresciau.6c00035)
Supplement: Supplementary file 1 [file tg6c00035_si_001.pdf]

Graphical Visualization Approach for Two-Dimensional Liquid Chromatography with Parallel Column Arrays

Deklin Parker[1], Samuel W. Foster[1], Tina Dahlseid[2], Dwight R. Stoll[2], and James P. Grinias[1,*]

[1]Department of Chemistry & Biochemistry, Rowan University, Glassboro, NJ 08028, United States

[2]Department of Chemistry, Gustavus Adolphus College, Saint Peter, MN 56082, United States

Supporting Information

SI1. Experimental Details

SI1.1. Reagents and Samples

Acetonitrile, formic acid, and ammonium bicarbonate were all obtained from… and used as obtained. A Milli-Q water purification system (Billerica, MA) was used to purify water in-house for use as a mobile phase. *Escherichia coli* (*E. coli*) digests were generously provided by Dr. Gregory Staples (Agilent Technologies).

SI1.2. E. coli *Preparation and Digestion*

E. coli lysate was digested with trypsin as described previously.[1]

SI1.3. Instrumentation

An Agilent 1290 Series LC instrument (Waldbronn, Germany) was used for this experiment. First- and second- dimension pumps (both Model G7120A) both include 35 µL JetWeaver mixers and columns for both dimensions were placed in a thermostated column compartment (Model G7116B). A diode array detector (Model G4212A) with a low dispersion flow cell (G4212-60038) was used after the first dimension. Active solvent modulation between the two dimensions was achieved using an Agilent valve interface (5067-4266). MS detection after the second dimension utilized a QTOF-MS (G6549A) with a JetStream ESI source. Both Agilent Chemstation (with 2D-LC add-on) and MassHunter software packages were used for system control and data analysis.

SI1.4. Columns

The first dimension of the experimental 2D-LC separation used a 150 mm x 2.1 mm i.d. Poroshell HPH-C18 column with 1.9 µm particle size. Two columns were tested in the second dimension. The first column was a 50 mm x 2.1 mm i.d. Agilent Zorbax SB-C18 column with 3.5 µm particle size. The second column was a 50 mm x 2,1 i.d. Agilent Zorbax Bonus-RP column with 3.5 µm particle size (all from Agilent Technologies).

SI1.5. Separation Conditions

^{1}D Conditions

Column: 150mm x 2.1mm Agilent Poroshell HPH-C18, 1.9µm

Flow Rate: 0.055 mL/min

Temperature: 35ºC

Mobile Phase A: 10 mM ammonium bicarbonate pH 9.5

Mobile Phase B: ACN

Gradient: 2-4.5-30-80-2-2% B in 1-2.5-50-55-55.01-65 min

Injection Volume: 10 µL

Sample: 1 µg/µL E.coli digest in 0.1% formic acid in 98:2 water:ACN

Detection: DAD (254, 214, 280 nm; 40 Hz)

^{2}D Conditions

Column: 50mm x 2.1mm Agilent Zorbax SB-C18, 3.5µm or

50mm x 2.1mm Agilent Zorbax Bonus-C18, 3.5µm

Flow Rate: 2 mL/min

Temperature: 60 ºC

Mobile Phase A: 0.1% Formic acid in water

Mobile Phase B: ACN

Gradient: 2-12.29-12.29%B in 0-0.01-0.68 min (shifting gradient; see below)

Equilibration Time: 0.06 min

Loops: On position 1 of both decks, 40 µL

Flush Sample Loop: 1.5 times (0.1 min)

Modulation Time: 0.8 min, 100% loop filling

Analysis Time: 0.68 min

ASM Factor: 2 (340 mm x 0.12 mm i.d. capillary)

Detection: QTOF (10 spectra/s)

^{2}D Gradient Shift Tables

**Gradient Shift ^{1}D Time for 0.01 min point in ^{2}D gradient**	**B [%]**
0.00	12.29
4.31	12.12
13.32	4.55
49.87	19.37
55.87	25.77
56.37	27.12
57.38	39.58

**Gradient Shift ^{1}D Time for 0.68 min point in ^{2}D gradient**	**B [%]**
0.00	12.29
4.31	12.29
15.33	24.76
19.33	27.79
29.34	34.53
57.38	41.60

SI2. Software Operation Guide

SI2.1. Start-Up Protocol

1. Create a Python file (main_poisson.py – can be created from the included text file) and download into the Python Integrated Development Environment (IDE) and then begin the data plotting process by selecting run (green arrow).

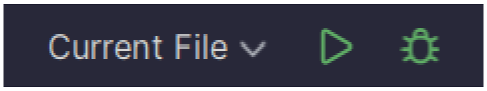

2. A small window (shown below) will appear for user-selected options: the desired number of peaks, plate count, the length of time of each second dimension separation (fraction rate), and the chromatographic void time.

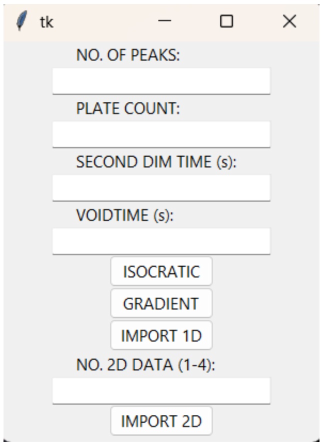

3. ISOCRATIC or GRADIENT operation modes can be selected for 1D and 2D simulations *(Section SI2.4*).

4. To import 1D data instead of simulate chromatograms, the IMPORT 1D button will allow the user to plot a 1D chromatogram (*Section SI2.2*). To import 2D data, the used can select how many 2D sets of data (*i.e.* number of columns in second dimension) will be plotted before selecting the IMPORT 2D button (*Section SI2.3*).

SI2.2. Unidimensional LC Data Import

1. Click the IMPORT 1D button and a file selection prompt will appear. The software is designed to import comma-delimited .CSV files (typically from Microsoft Excel) that are formatted with the x-axis data (time) in Column A and the y-axis data (signal) in Column B.

2. Upon selection of the appropriate file, the chromatogram is graphed as an x-y line plot. The bottom of the plot includes a set of buttons to adjust the plot (see graphic below).

- **a.** Home – Resets the default view
- **b.** Left and Right Arrows – Undo and Redo buttons, respectively
- **c.** Cardinal Arrows – Panning
- **d.** Magnifying Glass – Zoom
- **e.** Sliders – Adjust graph/axes/font size
- **f.** Floppy Disk – Save plot image

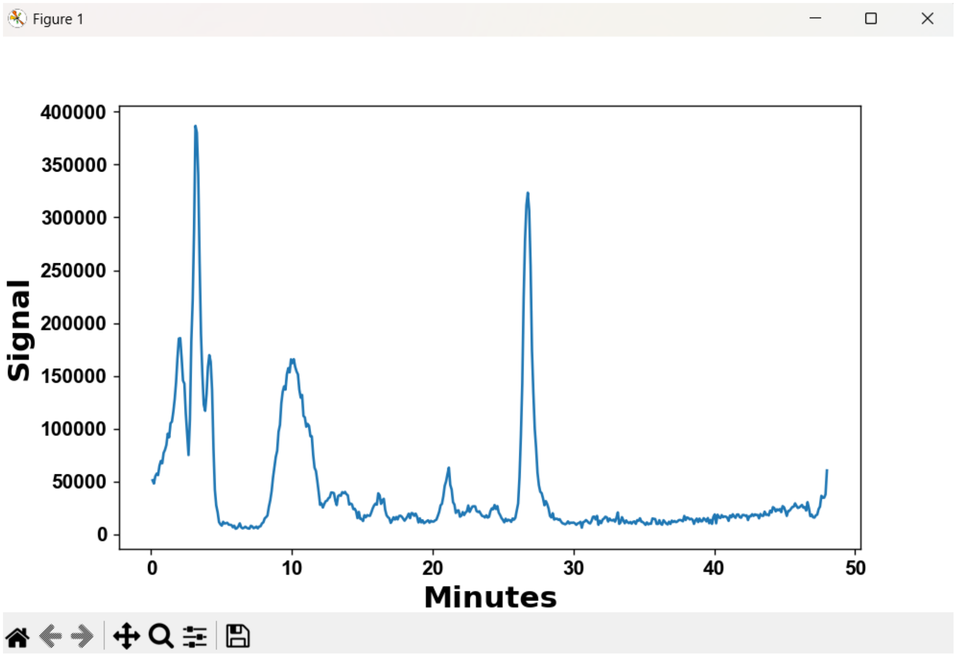

SI2.3. Two-Dimensional LC Data Import

Note: For proper functionality, the user must select the number of 2D data sets are being plotted simultaneously (between 1 and 4, with 1 being a standard 2D-LC method).

1. After selecting IMPORT 2D, a folder selection prompt will appear. If multiple columns are chosen, multiple folder selection prompts will appear. The folder(s) should be formatted such that each fraction is an individual chromatogram with the label 'Run_XYZ', where X, Y, and Z are numerals (*e.g.* Run_001)

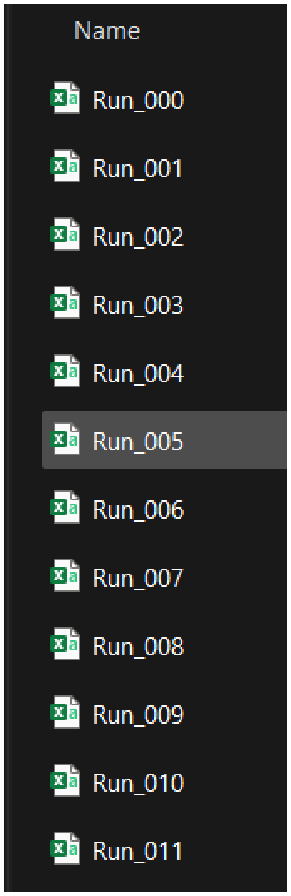

2. Upon selection of the appropriate folder, the 2D chromatogram is graphed as contour heatmap plot. The buttons in the top right provide the same functionality as described in *Section SI2.2*.

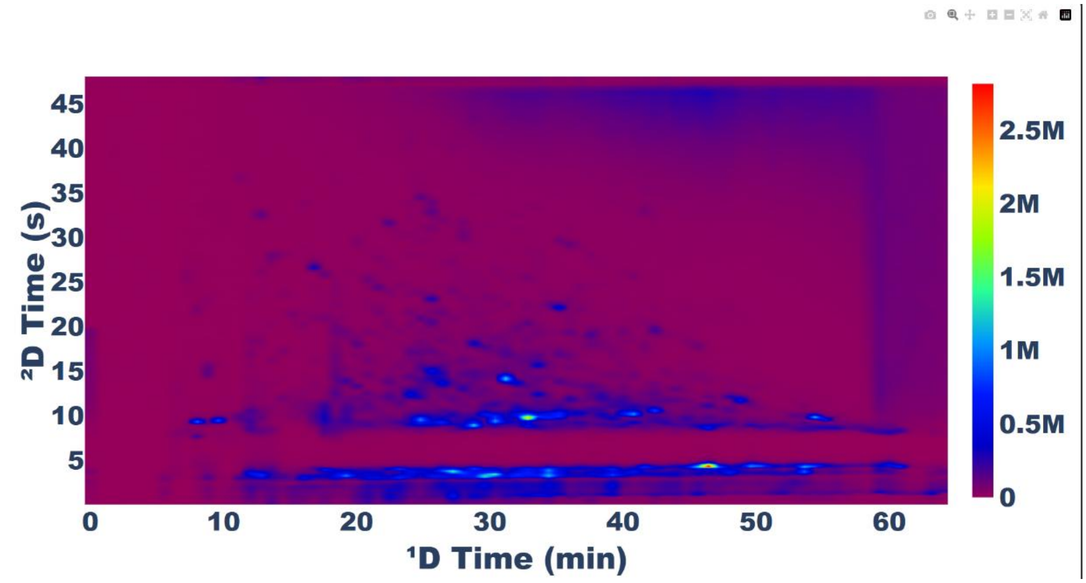

3. If more than one column in the second dimension is selected, a combined heatmap will open in a second window displaying the combination of the multiple datasets along with an estimation of separation space used (*Section 3*). A red marker can be placed on the plot to provide the breakdown of the contributing signal from each column (displayed as a vector on the color wheel to the right).

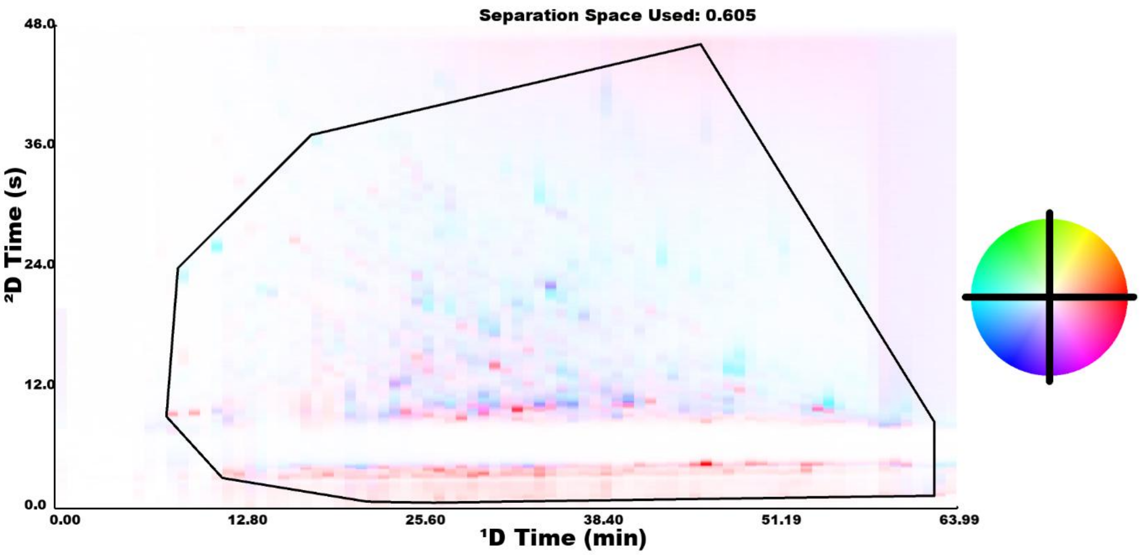

SI2.4. Simulation Inputs and Results

1. Input the desired parameters (*Section SI2.3*) into the user prompt. After parameters are entered, press either ISOCRATIC or GRADIENT to simulate the desired elution mode.

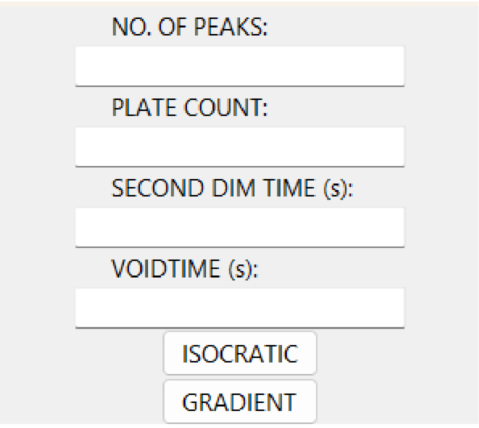

2. Once a method type is selected, multiple windows will open once the simulation is completed:

   a. A first dimension chromatogram with “Time” (seconds) on the x-axis and “Signal” on the y-axis (*Section SI2.2 Unidimensional LC Data Import*)

   b. Using an internet browser window, 2D chromatograms are generated. Four tabs are created showing four different second dimension separation simulations. (*Section SI2.3 Two-Dimensional LC Data Import*)

   c. A combined heatmap of the four second dimension separation simulations created in the previous step is generated. This shows the overlap between the generated data. (*Section SI2.3 Two-Dimensional LC Data Import*)

References

(1) Stoll, D. R.; Lhotka, H. R.; Harmes, D. C.; Madigan, B.; Hsiao, J. J.; Staples, G. O. High Resolution Two-Dimensional Liquid Chromatography Coupled with Mass Spectrometry for Robust and Sensitive Characterization of Therapeutic Antibodies at the Peptide Level. *J. Chromatogr. B Anal. Technol. Biomed. Life Sci.* **2019**, *1134–1135*, 121832. https://doi.org/10.1016/j.jchromb.2019.121832.
